# Saturation mutagenesis of selected residues of the α-peptide of the lantibiotic lacticin 3147 yields a derivative with enhanced antimicrobial activity

**DOI:** 10.1111/1751-7915.12041

**Published:** 2013-02-25

**Authors:** Des Field, Evelyn M Molloy, Catalin Iancu, Lorraine A Draper, Paula M O' Connor, Paul D Cotter, Colin Hill, R Paul Ross

**Affiliations:** 1 Department of Microbiology, University College CorkCork, Ireland; 2 Faculty of Food Science and Engineering, ‘Dunarea de Jos’, University of GalatiGalati, Romania; 3 Alimentary Pharmabiotic Centre, University College CorkCork, Ireland; 4 Teagasc Food Research Centre, Moorepark, Fermoy, Co.Cork, Ireland

## Abstract

The lantibiotic lacticin 3147 consists of two ribosomally synthesized and post-translationally modified antimicrobial peptides, Ltnα and Ltnβ, which act synergistically against a wide range of Gram-positive microorganisms. We performed saturation mutagenesis of specific residues of Ltnα to determine their functional importance. The results establish that Ltnα is more tolerant to change than previously suggested by alanine scanning mutagenesis. One substitution, LtnαH23S, was identified which improved the specific activity of lacticin 3147 against one pathogenic strain, *Staphylococcus aureus* NCDO1499. This represents the first occasion upon which the activity of a two peptide lantibiotic has been enhanced through bioengineering.

**Funding Information** Work in the authors' laboratory is supported by the Irish Government under the National Development Plan; by the Irish Research Council for Science Engineering and Technology (IRCSET); by Enterprise Ireland; and by Science Foundation Ireland (SFI), through the Alimentary Pharmabiotic Centre (APC) at University College Cork, Ireland, which is supported by the SFI-funded Centre for Science, Engineering and Technology (SFI-CSET) and provided P.D.C., C.H and R.P.R. with SFI Principal Investigator funding.

## Introduction

Lantibiotics [lanthionine-containing antibiotics (Schnell *et al*., 1988)] are a member of the family of antimicrobial peptides termed bacteriocins (Willey and van der Donk, 2007; Bierbaum and Sahl, 2009). In lantibiotics, dehydroalanine (Dha) and dehydrobutyrine (Dhb) residues are formed through the dehydration of serine and threonine respectively. The eponymous lanthionine (Lan) and β-methyllanthionine (MeLan) residues are enzymatically introduced when a covalent (thio-ether) bridge forms between a neighbouring cysteine and one of these unsaturated amino acids. These post-translational modifications confer structure and function to the previously inactive precursor peptide. Lantibiotics have been the subject of intensive studies as a result of their broad target range, potent activity and their potential as safe, natural food additives or as chemotherapeutic agents (Cotter *et al*., 2005b; Galvez *et al*., 2007; Piper *et al*., 2009a).

Lacticin 3147 is a lantibiotic produced by the food-grade bacterium *Lactococcus lactis* spp. *lactis* DPC3147. It is active against a variety of clinically significant Gram-positive organisms, including methicillin-resistant *Staphylococcus aureus* (MRSA), vancomycin-resistant *Enterococcus* strains (VRE) and penicillin-resistant *Pneumococcus*, in addition to foodborne pathogens such as *Listeria monocytogenes* and *Bacillus cereus* (Lawton *et al*., 2007b; Piper *et al*., 2009b; Carroll *et al*., 2010). Lacticin 3147 is a two peptide lantibiotic and thus both peptides, Ltnα and Ltnβ, are required for full antimicrobial activity (Wiedemann *et al*., 2006). Lacticin 3147 is active at single nanomolar concentrations through a dual mechanism in which Ltnα first interacts with the cell wall precursor lipid II to inhibit peptidoglycan synthesis. It is proposed that the resulting Ltnα : lipid II complex then interacts with Ltnβ to facilitate pore-formation (Wiedemann *et al*., 2006). An unusual feature of lacticin 3147 is the presence of three d-alanines (D-Ala) that are enzymatically derived from ribosomally introduced l-serines (Cotter *et al*., 2005c). Lacticin 3147 is one of only two examples of prokaryotic gene-encoded peptides in which such modified residues have been identified (Skaugen *et al*., 1994).

**Figure 1:**
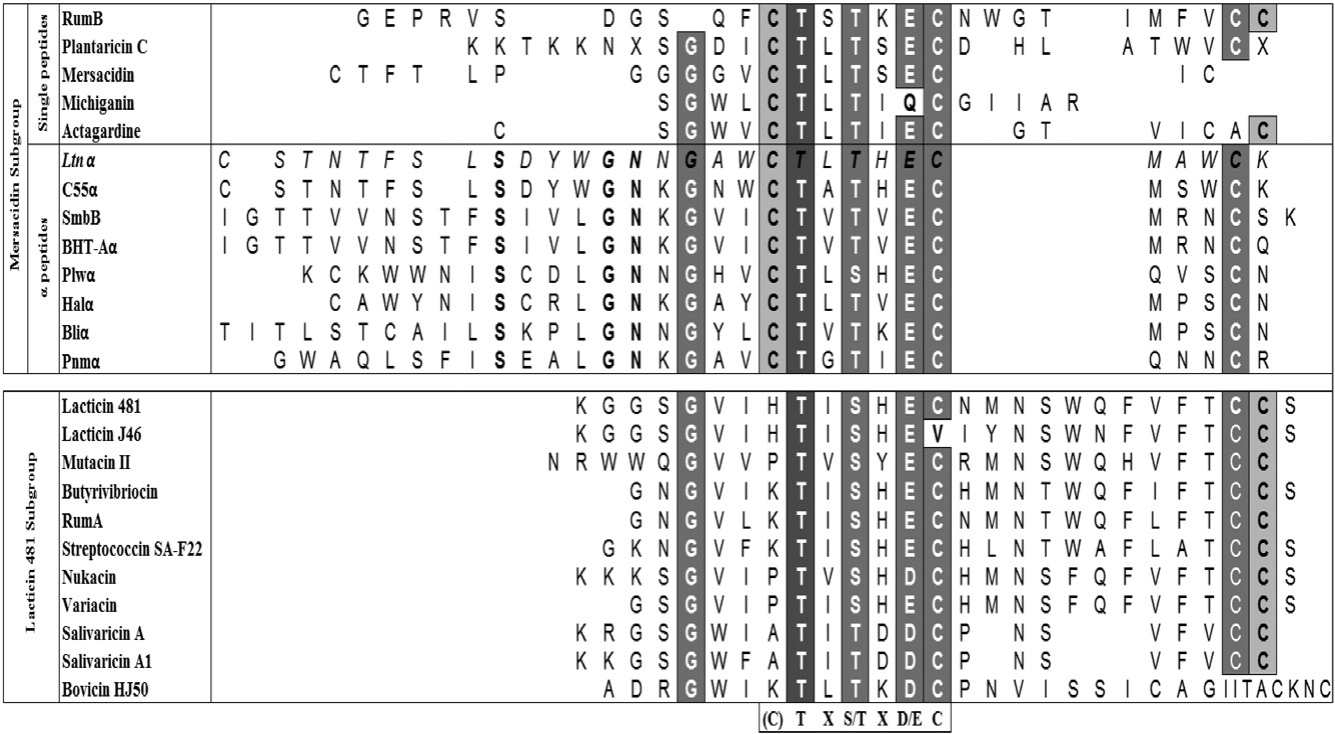
Alignment of the unmodified sequence of structural peptides of the mersacidin-like lantibiotic subgroup (Mersacidin Subgroup), which includes single-peptide members and the closely related α peptides of two peptide lantibiotics, and the lacticin 481-like lantibiotic subgroup (Lacticin 481 Subgroup). The sequence of Ltnα is italicized. Dark grey boxes indicate residues that are fully conserved across both subgroups, medium grey indicates residues that are highly conserved across both subgroups, while light grey indicates partially conserved residues. The putative lipid II binding motif conserved among the mersacidin and lacticin 481 lantibiotic subgroups (with the exception of (C), which is not found in the lacticin 481 subgroup) is given at the bottom of the alignment.

Two peptide lantibiotics remain relatively uncommon. To date only seven other lacticin 3147-like antimicrobials have been characterized; staphylococcin C55 (Navaratna *et al*., 1999), plantaricin W (Holo *et al*., 2001), Smb (Yonezawa and Kuramitsu, 2005), BHT-A (Hyink *et al*., 2005), haloduracin (McClerren *et al*., 2006; Lawton *et al*., 2007a), lichenicidin (Begley *et al*., 2009; Dischinger *et al*., 2009) and a predicted pneumococcin (Majchrzykiewicz *et al*., 2010). The α peptides of the two peptide lantibiotics cluster with the mersacidin-like peptides (Cotter *et al*., 2005a), with the exception of the enterococcal cytolysin (Cox *et al*., 2005). Despite their different overall structures, alignment of the mersacidin-like peptides with those from the more distantly related lacticin 481 subgroup (Fig. 1) reveals the presence of a conserved stretch of amino acids, (C)TXS/TXD/EC, a motif encompassing a conserved ring that has been suggested to comprise the core site for lipid II binding (Breukink and de Kruijff, 2006; Cotter *et al*., 2006). Significantly, this region represents the first point of contact between mersacidin and the bacterial cell and is thus believed to contain core amino acids that are essential with regard to the functionality of these peptides (Hsu *et al*., 2003).

Lantibiotics represent one possible solution to combat the emergence of multi-drug resistant strains (Boucher *et al*., 2009). The fact that lantibiotics are gene-encoded and ribosomally synthesized has facilitated the development of versatile expression systems capable of producing novel derivatives. This approach has generated information about the structure-function relationships of lantibiotics, as well as allowing one to screen for the enhancement of chemical and antimicrobial properties (Field *et al*., 2010a). A description of the role of each individual amino acid or domain will be required for a rational approach to design new, improved lantibiotics. To this end, an attempt has been made to predict the identity of essential and variable residues across both peptides of lacticin 3147 using alanine scanning mutagenesis (Cotter *et al*., 2006). However, the consequences of changing a residue to alanine do not always reflect the overall tolerance/intolerance of a specific residue to change. To address this issue, saturation mutagenesis of the lipid II-binding component of lacticin 3147, i.e. Ltnα, was performed. We focused on the residues of Ltnα that are not involved in bridge formation and while this approach frequently confirmed the relevance of the alanine scanning approach with regard to the importance of specific amino acids, a limited number of substitutions were found to be tolerated at positions previously designated as being intolerant of change. We also noted that amino acids highly conserved across related lantibiotics are not necessarily inviolate. More significantly, we identified the first derivative of a two peptide lantibiotic with increased potency against a strain of clinical significance.

## Results and discussion

Saturation mutagenesis was performed using a PCR-based approach and a two-plasmid expression system was subsequently applied in generation of banks of Ltnα mutants (Field *et al*., 2007), with a particular focus on mutants that retained at least some bioactivity against the sensitive indicator strain *L. lactis* HP (Table 1). As the aim of the current study was to confirm whether individual residues are tolerant or intolerant of change, no attempt was made to distinguish between mutations that impact on production and those that impact on specific activity.

**Table 1:** Bioactivity of *L. lactis* MG1363 pOM44 pDF02 derivatives producing mutant Ltnα peptides as determined by triplicate deferred antagonism assays against the sensitive indicator strain *Lactococcus lactis* spp. *cremoris* HP

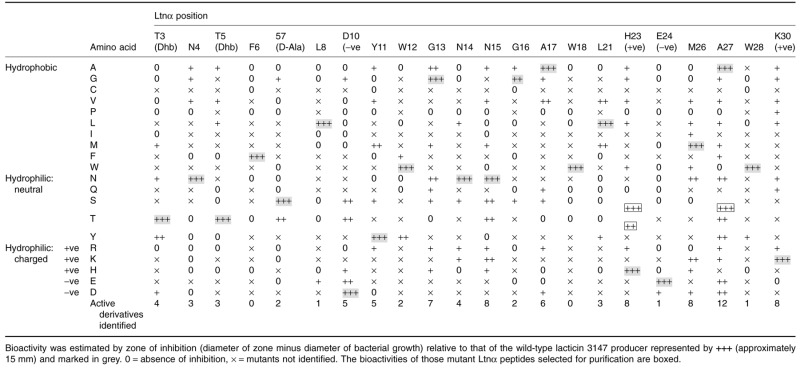

### Targeting of ‘essential’ residues in Ltnα for site-saturation mutagenesis

**Figure 2:**
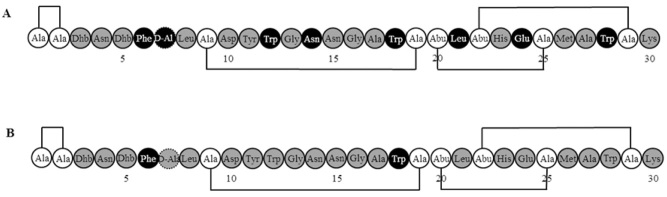
Tolerance of residues of Ltnα to change as determined by (A) alanine scanning, with the assumption that lack of bioactivity on substitution with alanine (or glycine in the case of a native alanine) indicates an immutable residue, while retention of bioactivity suggests that other residues could be tolerated at the specific position, and (B) saturation mutagenesis, with those that retain bioactivity only when the native residue is present designated as immutable residues, and those retaining bioactivity on substitution with one or more other residues classified as tolerant to change. Grey circles indicate tolerant positions while black circles indicate immutable positions. White circles represent residues involved in bridge formation, which were not targeted in this study.

The conversion of a number of Ltnα residues to alanine resulted in the abolition of bioactivity (Cotter *et al*., 2006); as such, these residues were designated as being ‘essential’ for bioactivity of lacticin 3147. These include residues proposed to be involved in the interaction with Ltnβ (F6, S7, W12, N14), putative lipid II binding residues (L21, E24) and two tryptophans (W18 and W28). Despite not being conserved (Fig. 1), the replacement of F6 and W12 with alanine was previously found to eliminate bioactivity (Cotter *et al*., 2006). Here saturation mutagenesis established that conservative substitutions are tolerated at position 12 (Fig. 2), with bioactivity decreasing relative to the size of the newly incorporated residue (Trp > Tyr > Phe; Table 1). However, a critical role was confirmed for F6 with respect to bioactivity (Cotter *et al*., 2006) (Table 1). Based on previous observations (Jing *et al*., 2003; Sanderson and Whelan, 2004), there is a likelihood that aromatic amino acids in membrane-acting peptides such as these are likely to be situated at the lipid-water interface and promote hydrophobic interaction with the cytoplasmic membrane. Thus, replacing the native residue with any amino acid other than another aromatic residue could be expected to have a detrimental effect on antimicrobial activity (Cotter *et al*., 2006).

Position S7 is subject to a two-step post-translational modification to form d-alanine (Cotter *et al*., 2005c). Despite the natural presence of an alanine at the corresponding location in Bliα and the fact that only Ltnα, and potentially Sacα (Suda *et al*., 2011), possess a d-alanine at this location (Fig. 1), an S7A mutant was previously found to be inactive (Cotter *et al*., 2005c; Cotter *et al*., 2006). Here we confirm the negative consequences of such a S7A change and note that many other substitutions also result in the elimination of bioactivity. However, in line with previous investigations, S7T (dehydrated to Dhb, data not shown) and S7G substitutions (Cotter *et al*., 2005c) were both found to result in active mutants (Table 1), confirming a limited tolerance to change (Fig. 2).

The previously generated N14A mutant lacked bioactivity (Cotter *et al*., 2006), in accordance with the complete conservation of N14 among lantibiotic α peptides (Fig. 1). This suggested a pivotal role for this residue in the synergistic interaction between both component peptides (Cotter *et al*., 2006). However, it is now apparent that some substitutions are tolerated to some degree, including replacements with positively charged residues arginine or lysine (Table 1).

Despite the variability of L21 across the mersacidin and lacticin 481 subgroups and the presence of alanine at the corresponding location in the closely related C55α (Fig. 1), a LtnαL21A mutant was previously found to be inactive (Cotter *et al*., 2006; O'Connor *et al*., 2007). Thus, this position was previously designated as being essential. However, site-saturation of L21 found that two conservative substitutions are tolerated, L21M and L21V, and perhaps surprisingly, a L21Y mutant retained a small amount of bioactivity (Table 1). The retention of bioactivity by the L21V mutant is notable in that it renders Ltnα more similar to other α peptides (SmbB, BHT-Aα and Bliα; Fig. 1). The results of saturation mutagenesis at positions N14 and L21 highlight the risk of relying solely on alanine scanning as an indication of tolerance to change (Fig. 2).

E24 is highly conserved among the mersacidin and lacticin 481 subgroups (Fig. 1) and LtnαE24A showed no bioactivity (Cotter *et al*., 2006). When the corresponding residues in mersacidin (Szekat *et al*., 2003), actagardine (Boakes *et al*., 2009) and Halα of haloduracin (Cooper *et al*., 2008) were substituted with Ala (and also to Gln in the case of HalαE22), all antimicrobial activity was also lost. HalαE22Q was also deficient with regard to the inhibition of peptidoglycan formation by the enzyme PBP1b, which uses lipid II as a substrate for glycan polymerization (Oman *et al*., 2011). Unsurprisingly, on saturation mutagenesis of LtnαE24, the only mutant to retain activity, albeit low levels, is one in which the negative charge at this position is maintained (E24D; Table 1). This is consistent with previous findings (Deegan *et al*., 2010) and renders the peptide more similar to many members of the lacticin 481 subgroup (Fig. 1). This observation contradicts a previous investigation that designated E24 as intolerant of change (Cotter *et al*., 2006) (Fig. 2). However, while a preference for the native glutamate is apparent, it can be said that a negatively charged residue at this position of Ltnα is of critical importance.

Despite their variable nature across the mersacidin-like peptides (Fig. 1), bioactivity was abolished when W18 and W28 were converted to alanine (Cotter *et al*., 2006). It was noted that on saturation mutagenesis of position W18, none of the Ltnα mutants identified retained detectable bioactivity (Table 1). In contrast, a W28Y mutant retained bioactivity, albeit at a reduced level compared to the wild-type, in disagreement with the anticipated intolerance to change at this location (Cotter *et al*., 2006) (Table 1; Fig. 2).

### Targeting of ‘variable’ residues in Ltnα for site-saturation mutagenesis

Many Ltnα residues were classified as variable in that they could be altered to alanine (or glycine in the case of native alanines) without resulting in complete loss of bioactivity (Cotter *et al*., 2006). These include the N-terminal variable residues (T3, N4, L8, T5), ring B variable residues (D10, Y11, G13, N15, G16, A17) and C-terminal variable residues (H23, M26, A27, K30). It should be noted that some changes which were previously found to be tolerated (Cotter *et al*., 2006) did not yield bioactive strains on this occasion, presumably as a consequence of the reduced activity associated with the expression system used in this study.

Residues T3 and T5 of Ltnα are both dehydrated to Dhb in mature lacticin 3147. Given the degree to which the previously generated T3A mutant retained bioactivity (Cotter *et al*., 2006), coupled with the fact that a threonine at this position is not conserved across the α peptide group (Fig. 1), it is perhaps not surprising that four bioactive mutants, T3M, N, Y and D, were identified on saturation mutagenesis of this residue (Table 1). Interestingly, none of these substitutions were residues present at the corresponding locations in other members of the group (Fig. 1). The retention of low levels of bioactivity by T5A, which renders the peptide more similar to Bhaα, was replicated (Cotter *et al*., 2006), and two additional bioactive mutants were identified (T5V, L; Table 1). Significantly, T5V rendered Ltnα more similar to the related α peptides SmbB and BHT-Aα (Fig. 1).

Because some activity was retained upon conversion of N4 to alanine (Cotter *et al*., 2006) and the residue at this position varies across the α peptide group (Fig. 1), N4 was previously categorized as being non-essential with respect to the bioactivity of lacticin 3147. Three active mutants were identified (Table 1), two of which involved substitutions which rendered the peptides more similar to other α peptides (A, Pnmα; V, SmbB and BHT-Aα) (Fig. 1). Similarly, L8 is variable across the group and a L8A mutant previously displayed bioactivity (Cotter *et al*., 2006). In accordance with this, an additional bioactive mutant was detected (L8E; Table 1).

Previous mutagenesis of the residues within ring B of Ltnα suggested that this region is tolerant of change in that six of the nine corresponding mutants retain bioactivity when altered to alanine (Cotter *et al*., 2006). The first of these residues, the negatively charged residue D10, is not conserved across the α peptide group (Fig. 1) and five active mutants were identified (Table 1). In particular, the retention of bioactivity following substitution with another negatively charged residue, D10E, was anticipated in light of the natural presence of a glutamate at the corresponding position of Pnmα (Fig. 1). Despite the non-production of a D10K mutant previously (Deegan *et al*., 2010), it seems that the presence of a negative charge here is not essential, given that a D10H mutant still retained some bioactivity.

In the case of four of the five aromatic residues in Ltnα (F6, W12, W18 and W28), conversion to alanine completely eliminated bioactivity (Cotter *et al*., 2006). The exception, Y11A, displayed greatly reduced bioactivity. This aromatic residue also varies across the α peptide group (Fig. 1). Indeed, five mutants with reduced bioactivities were identified (Table 1), including substitutions that rendered Ltnα more similar to other members of the group: Y11A (Pnmα), Y11V (SmbB, bhtA-alpha) and Y11R (Bhaα) (Fig. 1).

G13A was previously found to retain a considerable level of bioactivity (Cotter *et al*., 2006), even though glycine is conserved in all α peptides (Fig. 1). It was suggested that alanine alone, because of its similarity to the native glycine, could be tolerated (Cotter *et al*., 2006). However, saturation mutagenesis established the tolerance of the G13 residue to a variety of substitutions, ranging from other hydrophobic residues (G13A), to non-conservative hydrophilic neutral (G13N and G13Q) and charged residues (G13H and G13R) (Table 1). It should be noted that the bioactivity level of all G13 mutants was much reduced when compared to wild-type.

Residue G16 is even more highly conserved than G13, being fully conserved across both the mersacidin-like peptides (except RumB) and lacticin 481-like peptides (Fig. 1). This hyper-conserved nature, and the reduced and absent bioactivity of G16A (Cotter *et al*., 2006) and G16E (Field *et al*., 2007), respectively, suggested that G16 is less tolerant of change than its G13 counterpart. This was indeed the case as only one additional substitution retained detectable bioactivity (G16S, in which the Ser residue remains unmodified; data not shown) (Table 1). Similarly, on saturation mutagenesis of the corresponding glycine in mersacidin (G9), only three bioactive mutants were identified, including G9A and G9S (Appleyard *et al*., 2009). The corresponding position in nukacin ISK-1 (G5) has been shown by saturation mutagenesis to be essential to bioactivity (Islam *et al*., 2009).

Residues N15 and the previously discussed N14 are noteworthy due to the contrasting consequences on conversion to alanine, with N15A retaining significant bioactivity (Cotter *et al*., 2006). Saturation mutagenesis of N15 revealed eight mutants that retained bioactivity (Table 1), including the previously described N15A. In line with previous studies (O'Connor *et al*., 2007; Deegan *et al*., 2010), an N15K mutant, which more closely resembles the related C55α, SmbB, BHT-Aα, Halα and Pnmα peptides (Fig. 1), exhibited relatively high levels of bioactivity (Table 1). A N15S substitution that alters Ltnα to more closely resemble rumB, plantaricin C, michiganin and actagardine and many of the lacticin 481 peptides (Fig. 1) was also tolerated (Table 1).

Residue A17 is expected to be amenable to substitution based on its variation among related peptides (Fig. 1) and the fact that high activity was observed on substitution with glycine (Cotter *et al*., 2006). In keeping with this hypothesis, site-saturation at this position yielded a number of active mutants (Table 1). Surprisingly, no residues found at the corresponding locations in other group members were identified. We did not detect a previously described mutation, A17N, that makes Ltnα more closely resemble Sacα and which has little impact on bioactivity (O'Connor *et al*., 2007).

Although M26 is conserved in six out of eight α peptides (Fig. 1), the retention of some activity on substitution with alanine led to its classification as a residue that is amenable to change (Cotter *et al*., 2006). Indeed, following saturation mutagenesis, many active substitutions were identified (Table 1), including M26L that more closely resembles some members of the lacticin 481 subgroup. While an M26I mutant was only slightly active, mutation of the corresponding residue (V22) in nukacin ISK-1 to isoleucine resulted in a variant with increased potency (Islam *et al*., 2009).

In keeping with its designation as a variable residue (Cotter *et al*., 2006), and its non-conserved nature (Fig. 1), position A27 was found to be very tolerant of change when subjected to site-saturation mutagenesis (Table 1) (Cotter *et al*., 2006). In fact, site-saturation mutagenesis of LtnαA27 yielded the greatest number of bioactive mutants. A change to arginine, which is found in the equivalent position in SmbBα and BHT-Aα, resulted in a mutant that retained much of its bioactivity. A change to valine, which renders the peptide more similar to Plwα, had a more damaging impact. The identification of an A27S variant was interesting given that previous attempts to construct this mutant in order to generate a derivative of Ltnα that more closely resembled C55α were unsuccessful (O'Connor *et al*., 2007). We established that this mutant retained close to wild-type levels of bioactivity (Table 1), and like A27T, remained in an unmodified form (data not shown). The A27S variant also displayed levels of bioactivity comparable to those of the wild-type against *S. thermophilus* NCDO2525 and *L. lactis* AM2 (data not shown). A27S was purified in order to determine its specific activity (see below).

Ltnα has a net neutral charge (two positive residues, H23 and K30; and two negative; D10 and E24). While alanine substitution of the negatively charged residues had a relatively major impact, changing the positively charged amino acids had a lesser effect (Cotter *et al*., 2006). Indeed, a previously described derivative substituting alanine for both positive residues still retained considerable bioactivity (Deegan *et al*., 2010). The H23 location also merited attention by virtue of being the only position in the region of Ltnα within the predicted lipid II binding domain (residues 19–25), which retained bioactivity on conversion to alanine. Furthermore, both H23 and K30 are variable across the mersacidin-like peptides (Fig. 1). Saturation mutagenesis indicated many permissible substitutions for these positively charged residues (Table 1). In two instances they were replaced by other positively charged amino acids (K30R and H23R). Mutants where the substitution mimics a natural variation between Ltnα and the other α peptides (Fig. 1) were identified, namely H23V (SmbB, BHT-Aα and Halα), K30N (Plwα, Halα and Bliα) and K30Q (BHT-Aα). Although it has previously been established that both H23D- and K30D-producing strains are bioactive (Deegan *et al*., 2010), these mutants were not identified in the current study, suggesting that they were not created or were not among those tested.

It was apparent that Ltnα H23S- and H23T-producing mutants retained close to wild-type levels of bioactivity against *S. aureus* NCDO1499, a clinical isolate involved in bovine mastitis, *S. thermophilus* NCDO2525 and *L. lactis* AM2 (data not shown). As a consequence of this bioactivity, coupled with our inability to detect these peptides by CMS, it was postulated that production of these peptides may be reduced and thus that specific activity may be relatively high. On that basis, the H23T and H23S peptides were selected for purification and further analysis. Following purification, masses of 3269 and 3255 Da were ascertained for H23T and H23S, respectively, indicating that both hydroxyl residues remained in an unmodified form. Significantly, a serine residue is naturally present at the corresponding positions in both mersacidin and plantaricin C (Fig. 1), which in the case of mersacidin is known to be modified to Dha.

### Peptide purification and specific activity studies

Reverse Phase-High Performance Liquid Chromatography (RP-HPLC) of H23S, H23T and A27S confirmed peptides of the expected mass, with the exception of an additional peak corresponding to 3285 Da for H23T, which was indicative of oxidation. This occurred despite the use of a variety of strategies designed to minimize this phenomenon. As a result its specific activity could not be accurately assessed. Accordingly, only purified H23S and A27S were utilized for specific activity studies.

The MICs of Ltnα A27S against *L. lactis* AM2 and *S. thermophilus* NCDO2525, both alone and when combined with Ltnβ, are higher than those of Ltnα and the wild-type Ltnα–Ltnβ combination (Table 2). H23S-Ltnβ had a MIC of 0.0313 μM against *S. thermophilus* NCDO2525, similar to the wild-type Ltnα–Ltnβ combination (Table 2). The MICs of the two Ltnα peptides were also identical when determined in isolation against *S. thermophilus* NCDO2525. It was noteworthy that the H23S variant alone is twofold more active than its wild-type counterpart against *L. lactis* AM2 but, when combined with Ltnβ, had a MIC the same as that of wild-type Ltnα–Ltnβ (Table 2). This is only the second example where one of the peptides of a two peptide lantibiotic exhibits increased solo specific activity relative to the parental molecule. The first such peptide, LtnβR27A, displayed a twofold increased specific activity against *L. lactis* HP when compared to Ltnβ alone (Deegan *et al*., 2010). However, in that case an eightfold decreased specific activity was observed when LtnβR27A was combined with its sister peptide Ltnα against HP. Most notably, further MIC-based investigations revealed that when LtnαH23S is combined with Ltnβ, their combined specific activity (0.25 μM) was twofold greater than the natural lacticin 3147 (0.50 μM) against *S. aureus* NCDO1499 (Table 2), thus making it the first example of the application of bioengineering to successfully enhance the activity of lacticin 3147, or indeed any two peptide lantibiotic. Prompted by this finding, further MIC-based analysis of LtnαH23S combined with Ltnβ against a wider selection of indicator strains including other staphylococcal isolates (*S. aureus* Newman, *S. aureus* Farm 1), enterococci (*E. casseliflavus* 5053, *E. faecium* 5119) and *L. lactis* HP revealed that in each case, a twofold decrease in specific activity compared to wild-type Ltnα–Ltnβ was apparent (Table 2). LtnαH23S alone did not show enhanced solo activity against any of the targets. Thus, although LtnαH23S exhibits enhanced specific activity, both alone and in combination with Ltnβ, this enhanced activity is very much a strain specific phenomenon.

**Table 2:** Minimum inhibitory concentration (MIC) of purified Ltnα, Ltnα–H23S and Ltnα–A27S alone, and in combination with equimolar concentrations of purified Ltnβ, against various Gram-positive organisms

Peptide	*L. lactis* AM2	*S. aureus* NCDO1499^a^	*S. thermophilus* NCDO2525	*S. aureus* Newman	*S. aureu*s Farm1	*E. casseliflavus* 5053	*E. faecium* 5119	*L. lactis* HP
Ltnα + Ltnβ	0.03125	0.500	0.03125	2.5	0.156	0.250	0.250	0.0156
LtnαH23S + Ltnβ	0.03125	**0.250**	0.03125	5.0	0.312	0.500	0.500	0.0313
LtnαA27S + Ltnβ	0.062	ND	0.0625	ND	ND	ND	ND	ND
Ltnα	0.937	1.875	0.937	> 10	10	> 3.75	3.75	0.937
LtnαH23S	**0.468**	1.875	0.937	> 10	> 10	> 3.75	> 3.75	1.875
LtnαA27S	1.874	ND	3.784	ND	ND	ND	ND	ND

a Clinical mastitis isolate.

Values given are identical results from three independent determinations (μM). Those values in bold represent MICs that are improved relative to that of the wild-type against the relevant strain.

ND, not determined.

## Conclusion

Only a small number of bioengineered lantibiotics had been created prior to 2005, including derivatives with enhanced antimicrobial activity (Liu and Hansen, 1992; Kuipers *et al*., 1996; Wiedemann *et al*., 2001; Yuan *et al*., 2004; Rink *et al*., 2007), derivatives with enhanced properties including improved solubility and stability (Liu and Hansen, 1992; Rollema *et al*., 1995; Yuan *et al*., 2004), or ones which enabled researchers to gain an appreciation of structure/function relationships (Chan *et al*., 1996; van Kraaij *et al*., [Bibr b52],[Bibr b51]; Chen *et al*., 1998; Wiedemann *et al*., 2001; Szekat *et al*., 2003). These pioneering studies suggested that lantibiotic peptides are quite adaptable and it was evident that further bioengineering-based approaches could be rewarding. Some recent examples have been successful with regard to the generation and identification of lantibiotic derivatives with improved antimicrobial and/or physicochemical properties (Rink *et al*., 2007; Field *et al*., [Bibr b21],[Bibr b19]; Appleyard *et al*., 2009; Islam *et al*., 2009; Field *et al*., 2010b; Rouse *et al*., 2012).

The two peptide lantibiotics have been the subject of much interest as they offer many possibilities with respect to the design of new, and possibly more potent, antimicrobials. To facilitate the rational design of such peptides, we performed saturation mutagenesis on one of the two lacticin 3147 peptides. There are already encouraging signs that Ltnα would make an excellent candidate for bioengineering considering the significant number of residues (16/30) that retained bioactivity following alanine scanning mutagenesis (Cotter *et al*., 2006), and the fact that it can function in combination with the β peptide from another two peptide lantibiotic (O'Connor *et al*., 2007). This flexibility is coupled with the fact that the involvement of two peptides facilitates the examination of distinct functional domains in isolation (Morgan *et al*., 2005). While both Ltnα and Ltnβ each possess solo activity, Ltnα is significantly more active than Ltnβ. Thus, Ltnα derivatives can be more easily assessed in isolation, as well as in combination with Ltnβ. It has been speculated that once the basis of the mutual interaction between the α and β peptides is revealed, theoretically the α peptide could be directed to other more strain-specific targets than lipid II (Breukink and de Kruijff, 2006), while continuing to interact with the β peptide to facilitate pore formation.

To this end, site-saturation mutagenesis was performed on all residues of Ltnα other than those involved in bridge formation, facilitating a more comprehensive determination of the tolerance of Ltnα to change than that provided by alanine scanning (Fig. 2). It was apparent that a number of positions in particular were more amenable to change (N14, L21) than was previously predicted (Cotter *et al*., 2006). Furthermore, a limited number of mostly conservative changes were tolerated at positions previously designated as intolerant (S7, W12, E24 and W28) (Cotter *et al*., 2006). Significantly, despite the conserved nature of positions G13, G16 and M26, it was found that within lantibiotics a high degree of conservation does not necessarily mean that change at this location is not tolerated.

Additionally, during this process, a H23S substitution was found to improve the specific activity of lacticin 3147 against a strain of *S. aureus* responsible for bovine mastitis, and that of the Ltnα peptide alone against *L. lactis* AM2. While the bioengineering of lantibiotics has produced some successes and the activity of a number of one peptide lantibiotics has been enhanced, this is the first description of a bioengineered two-peptide lantibiotic with an improved specific activity. The fact that such enhanced combinations have not been described previously most likely stems from the requirement for two peptides to act synergistically for full activity. This imposes a greater structural constraint on each peptide, and thus alterations made to enhance the interaction of the α peptide with its cell target for instance may have a negative impact on its ability to function synergistically with the β peptide. One might have predicted that the H23S alteration could enhance lipid II binding as a consequence of the peptide more closely resembling mersacidin, whose activity is solely based on lipid II binding without pore formation. However, the fact that the solo activity of Ltnα H23S against *S. aureus* is not improved confirms that the enhanced activity is dependent on the presence of Ltnβ. We speculate that this change must either improve the Ltnα–Ltnβ interaction at the target site or that an enhanced Ltnα–lipid II interaction may require a Ltnβ-induced conformational change. Future work will focus on the elucidation of the mechanistic basis for the strain-specific enhanced activity of lacticin 3147 H23S relative to lacticin 3147.

In summary, through the study of > 200 mutants, this systematic mutagenesis has provided significant information on the key residues that contribute to the bioactivity of lacticin 3147, which should prove valuable for the rational design of novel lantibiotics with improved properties. Furthermore, while the vast majority of mutants were less potent, the high number of derivatives that were produced in this study can also be interpreted as a test of the *in vivo* promiscuity of the enzymatic machinery, showing that the biosynthetic pathway of lacticin 3147 has a relatively relaxed specificity when it comes to mutants of Ltnα. Perhaps most importantly, a Ltnα–H23S change was found to improve the specific activity of lacticin 3147 against a strain of *S. aureus*, representing the first instance in which an enhanced bioengineered derivative of a two peptide lantibiotic has been identified.

## Experimental procedures

### Bacterial strains and growth conditions

Bacterial strains and plasmids used in this study are listed in Table S1. *L. lactis* and *Enterococcus* strains were grown in M17 broth (Oxoid) supplemented with 0.5% glucose (GM17) or GM17 agar at 30°C and 37°C respectively. *Escherichia coli* was grown in Luria–Bertani broth with vigorous shaking or agar at 37°C. *S. aureus* strains were grown in Mueller-Hinton broth (Oxoid) at 37°C. *S. thermophilus* NCDO2525 was grown in Litmus Milk (Difco BD, USA) before routine subculturing in M17 broth supplemented with 0.5% lactose (LM17) at 37°C. Chloramphenicol and tetracycline were used at 5 and 10 μg ml^−1^, respectively, for *L. lactis* (unless otherwise stated) where required and at 20 and 10 μg ml^−1^, respectively, for *E. coli.* Xgal (5-bromo-4-chloro-3-indolyl-β-D-galactopyranoside) was used at a concentration of 40 μg ml^−1^.

### Site-saturation mutagenesis

Oligonucleotide pairs (Table S2) were designed to replace each target *ltnA1* codon with the NNK triplet, which should result in the substitution of the relevant residue with all 19 possible alternatives (Cwirla *et al*., 1990; Scott and Smith, 1990). Plasmid pDF01 was used as template DNA for saturation mutagenesis and PCR amplification was performed as previously described (Field *et al*., 2008). Following plasmid amplification and introduction into the intermediate *E. coli* MC1000 host, plasmid DNA from a pooled bank of pDF01 derivatives (each corresponding to a targeted amino acid) was isolated using a Roche High Pure Plasmid Isolation Kit. DNA sequence analysis with pCI372FOR (MWG Biotech, Germany) confirmed randomization at the relevant codon. PbacA1A2 (containing bioengineered *ltnA1* genes, the partner *ltnA2* gene and the associated promoter region Pbac) was re-amplified using the primers pPTPLA1A2FOR and pPTPLA1A2REV and template DNA isolated from the individual mutagenized pDF01 pools. Amplified products were purified as before, digested with BglII and XbaI (Roche), ligated with similarly digested and shrimp alkaline phosphatase (Fermentas)-treated pPTPL and introduced by electroporation into *E. coli* MC1000. Transformants were pooled and stored in 80% glycerol at −20°C. Plasmid DNA isolated from each mutant bank was introduced by electroporation into the strain *L. lactis* MG1363 pOM44 to facilitate expression of the bioengineered Ltnα peptide (in the presence of unaltered Ltnβ) for further analysis. A total of 144 transformants were chosen at random and inoculated into 96-well plates containing GM17 chloramphenicol and tetracycline (5 μg ml^−1^ each), incubated overnight and stored at −20°C after addition of 80% glycerol. Mutants were identified by MS analysis and, in instances where the nature of the change remained ambiguous after MS or a peptide could not be detected, sequencing with TETK P1. All bioactive derivatives in each bank were identified. Ten representative inactive derivatives were chosen from each bank for further analysis, with loss of activity attributed to the particular substitution, an insertion, numerous mutations or the introduction of a stop codon. Varying levels of success were observed in the identification of unique inactive derivatives. Steps were taken to ensure that the companion peptide was unmutated (by MS and/or sequencing) in all cases.

### Matrix-assisted laser desorption/ionization time-of-flight (MALDI-TOF) mass spectrometry (MS)

Colony mass spectrometry (CMS) was performed with an Axima TOF^2^ MALDI-TOF mass spectrometer (Shimadzu Biotech, Manchester, UK) as previously described (Field *et al*., 2010b). For purified peptide, a small amount of lyophilized peptide resuspended in 70% IPA 0.1% TFA was used for analysis.

### Bioassays for antimicrobial activity

Deferred antagonism assays were performed as previously described (Field *et al*., 2007). For high throughput screening of the Ltnα site-saturation banks against *L. lactis* HP, deferred antagonism assays were performed by spotting strains using a 96-pin replicator (Boekel) on GM17 agar plates. Zone size was measured with callipers and calculated as the diameter of the zone of clearing minus the diameter of bacterial growth.

Minimum inhibitory concentration determinations were performed as described previously (Wiedemann *et al*., 2006), with incubation for 16 h at 30°C (*L. lactis*) or 37°C (*S. aureus*, *S. thermophilus* and *Enterococci*). The MIC was read as the lowest peptide concentration causing inhibition of visible growth.

### RP-HPLC purification of lacticin 3147 and Ltnα derivatives

Reverse phase-high performance liquid chromatography (RP-HPLC) was used to obtain pure lacticin 3147 and Ltnα derivatives as previously described (Suda *et al*., 2011).

## Supporting information

Table S1 Strains and plasmids used in this study. UCC, University College Cork; NCDO, National Collection of Dairy Organisms.

Table S2 Oligonucleotides utilised in this study. Pho indicates 5′ phosphate. Boldface represents randomized nucleotides (N = A + C + G + T, K = G + T, M = A + C). Underlined sequences represent restriction sites.
