# Supplementary material for: Saturation mutagenesis of selected residues of the α-peptide of the lantibiotic lacticin 3147 yields a derivative with enhanced antimicrobial activity
Source: Microb Biotechnol. 2013 Feb 25;6(5):564–75. doi: 10.1111/1751-7915.12041 (PMC3918158; doi:10.1111/1751-7915.12041)
Supplement: Table S1 — Strains and plasmids used in this study. UCC, University College Cork; NCDO, National Collection of Dairy Organisms. [file mbt0006-0564-sd1.docx]

**Table S1.**

| **Strain/Plasmid** | **Relevant characteristics** | **Source/Reference** |
| --- | --- | --- |
|  |  |  |
| **Strains** | | |
| *L. lactis* MG1363 pOM44 | MG1363 harbouring pOM44 | (Cotter, et al., 2006) |
| *L. lactis* MG1363 pOM44 pDF02 | MG1363 harbouring pOM44 and pDF02 | (Field, et al., 2007) |
| *E. coli* MC1000 | Intermediate cloning host | UCC Culture Collection |
| *E. coli* MC1000 pPTPL | MC1000 harbouring pPTPL | (O'Driscoll, et al., 2004) |
| *L. lactis* spp *cremoris* HP | Lacticin 3147 sensitive indicator | UCC Culture Collection |
| *L. lactis* AM2 | Lacticin 3147 sensitive indicator | UCC Culture Collection |
| *S. thermophilus* NCDO2525 | Lacticin 3147 sensitive indicator | NCDO |
| *S. aureus* NCDO1499 | Lacticin 3147 sensitive indicator | NCDO |
| *S. aureus* Newman | Lacticin 3147 sensitive indicator | UCC Culture Collection |
| *S. aureus* Farm1 | Lacticin 3147 sensitive indicator | UCC Culture Collection |
| *E. casseliflavus* 5053 | Lacticin 3147 sensitive indicator | UCC Culture Collection |
| *E. faecium* 5119 | Lacticin 3147 sensitive indicator | UCC Culture Collection |
|  | | |
| **Plasmids** | | |
| pCI372 | CmR; High-copy number cloning vector | (Hayes, et al., 1990) |
| pOM44 | pCI372-*ltnEFIRM1TM2J* | (Cotter, et al., 2006) |
| pPTPL | TetR; lacZ; Low-copy number cloning vector | (O'Driscoll, et al., 2004) |
| pDF01 | pCI372 with *ltnA1A2* under associated promoter Pbac | (Cotter, et al., 2006) |
| pDF02 | pPTPL with *ltnA1A2* under associated promoter Pbac | (Field, et al., 2007) |
|  | | |

1 Cotter, P. D., Draper, L. A., Lawton, E. M., McAuliffe, O., Hill, C., and Ross, R. P. (2006) Overproduction of wild-type and bioengineered derivatives of the lantibiotic lacticin 3147, *Appl Environ Microbiol* **72**: 4492-4496.

2 Field, D., Collins, B., Cotter, P. D., Hill, C., and Ross, R. P. (2007) A system for the random mutagenesis of the two-peptide lantibiotic lacticin 3147: analysis of mutants producing reduced antibacterial activities, *J Mol Microbiol Biotechnol* **13**: 226-234.

3 O'Driscoll, J., Glynn, F., Cahalane, O., O'Connell-Motherway, M., Fitzgerald, G. F., and Van Sinderen, D. (2004) Lactococcal plasmid pNP40 encodes a novel, temperature-sensitive restriction-modification system, *Appl Environ Microbiol* **70**: 5546-5556.

4 Hayes, F., Daly, C., and Fitzgerald, G. F. (1990) Identification of the Minimal Replicon of Lactococcus lactis subsp. lactis UC317 Plasmid pCI305, *Appl Environ Microbiol* **56**: 202-209.
