# Supplementary material for: Saturation mutagenesis of selected residues of the α-peptide of the lantibiotic lacticin 3147 yields a derivative with enhanced antimicrobial activity
Source: Microb Biotechnol. 2013 Feb 25;6(5):564–75. doi: 10.1111/1751-7915.12041 (PMC3918158; doi:10.1111/1751-7915.12041)
Supplement: Table S2 — Oligonucleotides utilised in this study. Pho indicates 5′ phosphate. Boldface represents randomized nucleotides (N = A + C + G + T, K = G + T, M = A + C). Underlined sequences represent restriction sites. [file mbt0006-0564-sd2.docx]

**Table S2.**

| **Primer name** | **Sequence (5’-3’)** |
| --- | --- |
| ltnA1T3degFOR | Pho-GCGTGTAGT**NNK**AACACATTCTCGCTCAGTGATTACTGG |
| ltnA1T3degREV | GAATGTGTT**MNN**ACTACACGCACCAAATACATCTTCATC |
| ltnA1N4degFOR | Pho-TGTAGTACT**NNK**ACATTCTCGCTCAGTGATTACTGGGGA |
| ltnA1N4degREV | CGAGAATGT**MNN**AGTACTACACGCACCAAATACATCTTC |
| ltnA1T5degFOR | Pho-AGTACTAAC**NNK**TTCTCGCTCAGTGATTACTGGGGAAAT |
| ltnA1T5degREV | GAGCGAGAA**MNN**GTTAGTACTACACGCACCAAATACATC |
| ltnA1F6degFOR | Pho-GTACTAACACA**NNK**TCGCTCAGTGATTACTGGGGAAATAAC |
| ltnA1F6degREV | TCACTGAGCGA**MNN**TGTGTTAGTACTACACGCACCAAATAC |
| ltnA1S7degFOR | Pho-AACACATTC**NNK**CTCAGTGATTACTGGGGAAATAACGGG |
| ltnA1S7degREV | ATCACTGAG**MNN**GAATGTGTTAGTACTACACGCACCAAA |
| ltnA1L8degFOR | Pho- ACATTCTCG**NNK**AGTGATTACTGGGGAAATAACGGGGCT |
| ltnA1L8degREV | GTAATCACT**MNN**CGAGAATGTGTTAGTACTACACGCACC |
| ltnA1D10degFOR | Pho-TCGCTCAGT**NNK**TACTGGGGAAATAACGGGGCTTGGTGT |
| ltnA1D10degREV | TCCCCAGTA**MNN**ACTGAGCGAGAATGTGTTAGTACTACA |
| ltnA1Y11degFOR | Pho-CTCAGTGAT**NNK**TGGGGAAATAACGGGGCTTGGTGTACA |
| ltnA1Y11degREV | ATTTCCCCA**MNN**ATCACTGAGCGAGAATGTGTTAGTACT |
| ltnA1W12degFOR | Pho-TCAGTGATTAC**NNK**GGAAATAACGGGGCTTGGTGTACACTC |
| ltnA1W12degREV | CCGTTATTTCC**MNN**GTAATCACTGAGCGAGAATGTGTTAGT |
| ltnA1G13degFOR | Pho-GTGATTACTGG**NNK**AATAACGGGGCTTGGTGTACACTCACT |
| ltnA1G13degREV | GCCCCGTTATT**MNN**CCAGTAATCACTGAGCGAGAATGTGTT |
| ltnA1N14degFOR | Pho-ATTACTGGGGA**NNK**AACGGGGCTTGGTGTACACTCACTCAT |
| ltnA1N14degREV | CAAGCCCCGTT**MNN**TCCCCAGTAATCACTGAGCGAGAATGT |
| ltnA1N15degFOR | Pho-ACTGGGGAAAT**NNK**GGGGCTTGGTGTACACTCACTCATGAA |
| ltnA1N15degREV | CACCAAGCCCC**MNN**ATTTCCCCAGTAATCACTGAGCGAGAA |
| ltnA1G16degFOR | Pho-GGGGAAATAAC**NNK**GCTTGGTGTACACTCACTCATGAATGT |
| ltnA1G16degREV | GTACACCAAGC**MNN**GTTATTTCCCCAGTAATCACTGAGCGA |
| ltnA1A17degFOR | Pho-GAAATAACGGG**NNK**TGGTGTACACTCACTCATGAATGTATG |
| ltnA1A17degREV | AGTGTACACCA**MNN**CCCGTTATTTCCCCAGTAATCACTGAG |
| ltnA1W18degFOR | Pho-ATAACGGGGCT**NNK**TGTACACTCACTCATGAATGTATGGCT |
| ltnA1W18degREV | GTGAGTGTACA**MNN**AGCCCCGTTATTTCCCCAGTAATCACT |
| ltnA1L21degFOR | Pho-CTTGGTGTACA**NNK**ACTCATGAATGTATGGCTTGGTGTAAA |
| ltnA1L21degREV | CATTCATGAGT**MNN**TGTACACCAAGCCCCGTTATTTCCCCAGT |
| ltnA1H23degFOR | Pho-GTACACTCACT**NNK**GAATGTATGGCTTGGTGTAAATAAGTT |
| ltnA1H23degREV | GCCATACATTC**MNN**AGTGAGTGTACACCAAGCCCCGTTATT |
| ltnA1E24degFOR | Pho-CTCACTCAT**NNK**TGTATGGCTTGGTGTAAATAAGTTAAT |
| ltnA1E24degREV | AGCCATACA**MNN**ATGAGTGAGTGTACACCAAGCCCGCAATAA |
|  |  |

**Table S2.** (cont.)

| **Primer name** | **Sequence (5’-3’)** |
| --- | --- |
| ltnA1M26degFOR | Pho-CATGAATGT**NNK**GCTTGGTGTAAATAAGTTAATAACAAA |
| ltnA1M26degREV | ACACCAAGC**MNN**ACATTCATGAGTGAGTGTACACCAAGC |
| ltnA1A27degFOR | Pho-ATGAATGTATG**NNK**TGGTGTAAATAAGTTAATAACAAATTT |
| ltnA1A27degREV | TATTTACACCA**MNN**CATACATTCATGAGTGAGTGTACACCA |
| ltnA1W28degFOR | Pho-AATGTATGGCT**NNK**TGTAAATAAGTTAATAACAAATTTTTA |
| ltnA1W28degREV | ACTTATTTACA**MNN**AGCCATACATTCATGAGTGAGTGTACA |
| ltnA1K30degFOR | Pho-TGGCTTGGTGT**NNK**TAAGTTAATAACAAATTTTTAATTAAT |
| ltnA1K30degREV | TTATTAACTTA**MNN**ACACCAAGCCATACATTCATGAGTGAG |
| pCI372FOR | CGGGAAGCTAGAGTAAGTAG |
| TETK P1 | AGTCCGTTAAATCGACTG |
| pPTPLA1A2FOR | TCAGATCTTATATACAGAGTTACTA |
| pPTPLA1A2REV | TGTCTAGATAATTTCTGGAAAAAC |
|  |  |
